# Accuracy and empathy of AI-based conversational chatbots in response to temporomandibular dysfunction related queries

**DOI:** 10.1016/j.pecinn.2026.100463

**Published:** 2026-02-23

**Authors:** Maryam Shehab, Tanmoy Bhattacharjee, Hisham Mohammed, Prasad Nalabothu, Moosa Abuzayeda, Keyvan Moharamzadeh, Jahanzeb Chaudhry, Nader Nabil Fouad, Abdel Rahman Tawfik, Sabarinath Prasad

**Affiliations:** a Department of Orthodontics, Hamdan Bin Mohammed College of Dental Medicine, Mohammed Bin Rashid University of Medicine and Health Sciences, Dubai, United Arab Emirates; b Mohammed Bin Rashid University of Medicine and Health Sciences, Dubai, United Arab Emirates; c Discipline of Orthodontics, The University of Queensland, Australia; d Department of Pediatric Oral Health and Orthodontics, University Center for Dental Medicine UZB, Basel, Switzerland; e Department of Prosthodontics, Hamdan Bin Mohammed College of Dental Medicine, Mohammed Bin Rashid University of Medicine and Health Sciences, Dubai, United Arab Emirates; f Department of Oral Diagnostics and Surgical Sciences, Hamdan Bin Mohammed College of Dental Medicine, Mohammed Bin Rashid University of Medicine and Health Sciences, Dubai, United Arab Emirates; g Oral and Maxillofacial Radiology, College of Dentistry, City University Ajman, Ajman, United Arab Emirates; h Department of Oral Surgery, Hamdan Bin Mohammed College of Dental Medicine, Mohammed Bin Rashid University of Medicine and Health Sciences, Dubai, United Arab Emirates

**Keywords:** Temporomandibular disorders, Chatbots, Empathy, Conversational AI, Large language models, Health informatics

## Abstract

**Objective(s):**

To compare the accuracy and empathy of responses generated by artificial intelligence (AI)-based chatbots to commonly asked temporomandibular dysfunction (TMD)-related questions. Additionally, test the performance of an automated text-based empathy detection model against subject matter experts (SMEs) judgments.

**Materials and methods:**

TMD-related questions (*n* = 14) were developed by a multidisciplinary panel of SMEs and categorized into five clinical domains (Diagnosis and testing, Causes and aggravating factors, Symptoms and associated issues, Treatment options, and Management and prognosis). Free-tier implementations of three AI-based chatbots: ChatGPT GPT-3.5 (CG), Claude 3.5 Sonnet (CD), and DeepSeek R1 (DS) were prompted to generate responses to these questions. Responses were rated for accuracy based on the Accuracy of Information (AOI) index, and empathy using a 3-point scale by the SMEs (*n* = 8). To complement expert assessments, a Bidirectional Encoder Representations from Transformers (BERT)-based empathy detection model was trained on the Empathy in Textual Online Medical Exchanges (EPITOME) dataset and validated against SME ratings.

**Results:**

DS generated responses with the highest word count (573.6 ± 132.7); significantly more than CG (263.4 ± 63.5) and CD (186.6 ± 25.6). DS also had the highest accuracy across all clinical domains. Overall accuracy of the responses generated by the three chatbots was high. However, variations in accuracy based on clinical domain of the question were observed. Empathy assessments revealed moderate reliability (correlation ∼0.6) among SMEs. The BERT model showed strong concordance with SME judgments for high-empathy responses but demonstrated lower agreement for low-empathy categorizations.

**Conclusion:**

AI chatbots show promise in providing accurate information regarding TMDs, but their ability to convey empathy remains limited. The observed differences in accuracy and empathy among the three AI chatbots examined are based on a limited dataset and should therefore be interpreted with caution. Current AI chatbots represent an intermediate stage of development, demonstrating adequate technical proficiency while remaining constrained in addressing the humanistic dimensions of patient care. Although empathy detection models may inform future development, significant challenges in empathetic communication persist.

## Highlights

• AI-based conversational chatbots are accurate sources of information for temporomandibular disorder-related queries.
• Variability in accuracy was observed in certain clinical domains despite the high overall chatbot accuracy.
• For rating empathy in the chatbot responses, the correlation among subject matter experts (SMEs) was moderate.
• The AI model developed aligned well with SME judgments for high-empathy responses, but less so for low-empathy ones.
• Although AI chatbots provide accurate TMD information, empathetic delivery remains a challenge.

## 1 Introduction

AI-based chatbots are emerging as a new resource for health information, challenging the dominance of traditional online sources [1]. These conversational chatbots are designed to emulate human conversation, providing guidance and responses to patient inquiries [2]. They have shown utility in contexts involving privacy-sensitive or socially stigmatized health issues [3]. Ease of access to information from AI-based conversational chatbots about symptoms and potential treatments enables patients to make informed and prompt healthcare decisions.

The success of AI chatbots in healthcare hinges critically on digital health literacy and patient trust. While AI chatbot performance now exceeds that of traditional search engines and maintains strong alignment with professional guidelines, highlighting their promise as patient education tools [4], significant barriers remain. Patients still hesitate to rely on chatbots for medical advice due to concerns about accuracy and AI's ability to understand human emotion [5]. Nevertheless, when effectively implemented, accurate delivery of evidence-based information through these platforms can guide patients toward appropriate care and management strategies.

Owing to the lack of proper medical oversight, accuracy and reliability of online health-related information is questionable and misinformation is a concern [6]. Misinformation or incomplete answers could lead to inadequate self-care or delays in seeking proper treatment [7]. Although AI-based conversational chatbots have potential to offer readily accessible information, a gap remains in understanding the quality of chatbot responses, particularly in terms of accuracy. To address this gap, recent research efforts have focused on evaluating the accuracy of chatbot responses in various dental specialties including implantology [8], endodontics [9], restorative dentistry [10], and orthodontics [11,12].

While accuracy is important, certain health conditions require particular attention to both informational accuracy and emotional support. Empathetic communication from healthcare providers has a direct and measurable impact on pain perception and recovery, adherence to treatment plans, and patient satisfaction [[13], [14], [15], [16]]. Temporomandibular dysfunctions (TMDs) represent one such area where the psychosocial dimension of care is critical. TMDs encompass a range of conditions affecting the temporomandibular joint, masticatory muscles, and associated anatomical structures. Pain associated with TMDs can reduce overall well-being and limit quality of life [17,18]. Management approaches for TMDs have to address both the physical and psychological dimensions [19,20].

Given that effective TMD management requires both precise medical information and empathetic support to address the complex interplay of pain, functional limitations, and emotional distress, it becomes critical to evaluate AI chatbots on both dimensions simultaneously. However, recent research by Kula et al. that assessed chatbot responses in the context of TMD [21], was limited to evaluating the clinical accuracy of a single chatbot, without examining the emotional support aspect of the interaction. Current literature lacks comprehensive assessment of how well AI chatbots balance informational accuracy with empathetic communication in healthcare contexts, particularly for conditions like TMD that significantly impact quality of life. Hence, this study addresses the gap through a comparative analysis of three AI chatbots, evaluating both the accuracy and empathetic quality of their responses to TMD-related queries. A secondary objective of this study is to test a custom-trained AI model for detecting empathy in chatbot responses to TMD-related queries.

## 2 Materials and methods

**Fig. 1:**
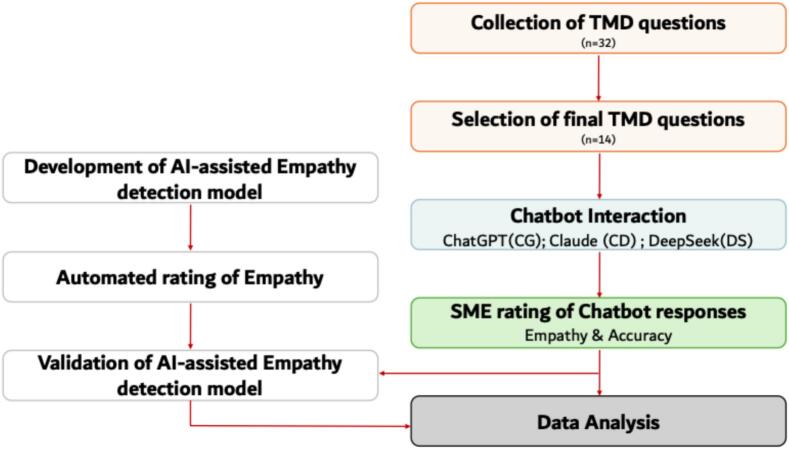
Schematic representation of the multi-phase methodology employed in the study. Note that the input stage of the study (light red) covers question collection and selection. The chatbot interaction stage (light blue) involved prompting the three chatbots (CG, CD, and DS). The evaluation stage (light green) includes SME ratings for empathy and accuracy along with automated empathy detection and validation followed by the final data analysis (light grey) stage. (For interpretation of the references to colour in this figure legend, the reader is referred to the web version of this article.)

The study was carried out in multiple phases. Fig. 1 outlines the different phases of the study.

### 2.1 Phase 1: question selection

The question selection process was guided by qualitative research principles. Four subject matter experts (SMEs), one each from orthodontics, prosthodontics, oral and maxillofacial surgery, and oral radiology, were independently invited to submit TMD-related questions frequently encountered in routine clinical practice. Two iterative refinement rounds were conducted. In the first round, the compiled questions were systematically reviewed to identify and remove duplicates and conceptually overlapping items, yielding a preliminary pool of unique questions (*n* = 32). In the second round, the four SMEs independently ranked the remaining questions according to clinical relevance and frequency of presentation based on real-world patient interactions. This consensus-driven prioritization resulted in a final set of questions (*n* = 14) that adequately captured all major dimensions of TMD clinical inquiry, thereby achieving thematic completeness. The final set of questions (n = 14) was then categorized into five domains based on widely accepted clinical criteria: (1) Diagnosis and testing, (2) Causes and aggravating factors, (3) Symptoms and associated issues, (4) Treatment options, and (5) Management and prognosis (Table 1).

**Table 1:** The list of clinical domains (*n* = 5) and TMD-related questions (*n* = 14) that were used to prompt the three chatbots (CD, CG, and DS) in this study.

Question No.	Domain	Question
1	C1: Diagnosis and testing	I have been diagnosed with temporomandibular disorder (TMD), but what exactly does that mean?
2		How is TMD diagnosed? Are there any special tests involved?
3	C2: Causes and aggravating factors	What could have caused my TMD?
4		I am experiencing a lot of stress at work recently. Does stress make TMD worse?
5	C3: Symptoms and associated issues	What are the most common symptoms of TMD that I should be aware of?
6		Can TMD cause other issues, like headaches or migraines? I've been experiencing both.
7		I have also been feeling some ear pain occasionally, could that be related to my TMD?
8	C4: Treatment options	What treatment options do I have to know about, now that I have TMD?
9		I have heard that removable occlusal plates help with TMDs?
10		I have heard that Botox injections are very helpful for managing my TMD. Is this true?
11		Will seeing an orthodontist to correct the way my teeth bite together cure me?
12	C5: Management and prognosis	How long does TMD usually last? Could it go away on its own, or do I need treatment?
13		Is TMD something I'll need to manage long-term, or is it curable?
14		Can I do anything to prevent TMD from getting worse?

### 2.2 Phase 2: AI chatbots

The selected questions were then input into the basic (free) version of three AI chatbots: ChatGPT GPT-3.5 (CG) (https://chat.openai.com/), Claude 3.5 Sonnet (https://claude.ai/) (CD), and DeepSeek R1(https://chat.deepseek.com/) (DS). The response randomness parameters of all of three AI chatbots were maintained at default settings. All chatbot queries were submitted using the same device (13-in. MacBook Air, Apple M2 chip, 8GB RAM, macOS Sequoia version 15.3.1), browser (Safari), and internet connection.

The selected questions were posed within the same day in the second week of February 2025 to control for temporal variability. The exact phrasing of the questions presented to the chatbots is presented in Table 1. All questions were presented in a patient-style phrasing, in a single-turn conversation and with no additional context or follow-up beyond the question.To reduce potential bias from prior interactions or algorithmic personalization, a new email account was created and used exclusively for the study. The responses generated by the chatbots were de-identified (emojis and unique chatbot formatting characteristics were removed) and compiled into a standardized form (Google Forms) under the same email account. The 14 questions were listed in the form in an anonymized manner to prevent evaluator bias regarding source. The order of chatbot responses in the form was randomly presented to the raters.

### 2.3 Phase 3: response rating

The form was then individually emailed to a rating panel consisting of eight dental specialists (2 orthodontists, 2 maxillofacial radiologists, 2 prosthodontists, and 2 maxillofacial surgeons). All raters were recognized SMEs with over a decade of clinical experience in their respective specialties. No calibration session or standardized examples were provided to SMEs prior to rating responses for accuracy and empathy. Raters applied their professional judgment based on the provided rating criteria without any prior alignment sessions. The SME ratings were collected anonymously through an online evaluation form, which did not request any identifying information other than professional role. The accuracy and empathy of the three chatbot responses were independently evaluated by the SMEs. To ensure confidentiality and data integrity, all collated ratings were stored in a secure, password-protected database accessible exclusively to the research team, in line with institutional policies.

#### 2.3.1 Accuracy assessment

The Accuracy of Information (AOI) index [11], which includes five dimensions: (1) Factual accuracy: alignment with evidence-based, up-to-date clinical information; (2) Corroboration: presence of supporting rationale or references; (3) Consistency: logical coherence within the response; (4) Clarity and Specificity: comprehensibility and level of detail; and (5) Relevance: how well the answer addresses the posed question, was used to evaluate the accuracy of chatbot responses. Each SME independently scored the chatbot responses across the five AOI dimensions (factual accuracy, corroboration, consistency, clarity and specificity and relevance) on a 0–2 scale (0 = Poor, 1 = Acceptable, 2 = Excellent), for a maximum AOI score of 10. The level of accuracy was categorized using a predefined scoring system (0–3 = low accuracy, 4–7 = moderate accuracy, and 8–10 = high accuracy).

#### 2.3.2 Empathy scoring and validation

Empathy in chatbot responses was evaluated using complementary approaches: SME rating and automated modelling. In the first approach, empathy was assessed as an additional item in the SME evaluation form. SMEs independently rated each chatbot response for empathetic content using a 3-point ordinal scale: 0 = no empathy, 1 = empathetic, and 2 = highly empathetic.

The second approach involved automated empathy detection using a custom-trained AI model. The process included data preparation, model training, performance evaluation, and validation against SME ratings. For training, the EPITOME dataset (∼10,000 post–response pairs from online mental health platforms) was used, with each response annotated for empathy on a 3-point scale (0 = no empathy, 1 = moderate empathy, 2 = high empathy) [22]. Preprocessing steps included lowercasing text, removal of punctuation, and substitution of all non-alphabetic characters with whitespace. A pretrained BERT (base-uncased) model (a type of language model that can be modified for specific tasks) was fine-tuned on the EPITOME dataset after preprocessing [23]. The dataset was split into training (80%) and validation (20%) sets. Training was performed with a batch size of 32 and the Adaptive Moment Estimation optimizer (ε = 1*e*^−8^), with a learning rate of 2*e*^−5^. Optimal performance was observed at epoch 4, achieving a minimum validation loss of 0.33 and a maximum validation accuracy of 0.74. The trained model was then saved and applied to score the chatbot responses. To prepare the chatbot output for automated scoring, the responses were segmented into individual sentences, and saved in *.csv* format for input into the trained model *via* the MATLAB programming platform. (Details of the codes used in the study are provided in Supplementary File). The scores generated by the AI model were subsequently compared with SME ratings to evaluate the validity of the automated empathy detection system.

### 2.4 Statistical analysis

Data were analyzed in SPSS version 28 (IBM, SPSS Inc., Chicago, IL, USA) and **s**tatistical significance was set at *p* < 0.05. Rater reliability among the eight SMEs was evaluated using the Intraclass Correlation Coefficient (ICC) with a two-way random-effects model. Reliability was interpreted using a standard benchmark scale [24]. The Shapiro-Wilk test was used to determine normality of the data, followed by one-way ANOVA with Dunn's test for multiple comparisons of the word counts in the responses generated by the different chatbots. Exploratory Pearson correlation coefficients were calculated to assess the relationship between word count and both the accuracy and empathy ratings.

## 3 Results

The three chatbots responded to all 14 questions, and a total of 42 answers were collected. CD provided answers with a relatively shorter word count (186.6 ± 25.6) than CG (263.4 ± 63.5) and DS (573.6 ± 132.7) (Table 2). Post-hoc analyses revealed statistically significant differences in word counts between all pairs of AI-based chatbots (*p* < 0.01 for CG *vs* CD; *p* < 0.0001 for CG *vs* DS and CD *vs* DS). Specifically, DS generated substantially more words than both CG (mean difference: −310.2; 95% CI: −391.1 to −229.3) and CD (mean difference: −387.0; 95% CI: −471.7 to −302.3), while CG generated more words than CD (mean difference: 76.79; 95% CI: 29.50–124.1). Domain-specific analysis highlighted significant differences between DS and both CG (mean difference: −410.8; *p* = 0.003) and CD (mean difference: −499.5; p < 0.0001) especially for the C4-Treatment options domain. However, no significant difference was observed between CG and CD in the C4-Treatment options domains (*p* = 0.15). All three chatbots frequently encouraged their users to see a dentist or medical professional for further details about their queries.

**Table 2:** Domain-wise distribution of accuracy scores (median and inter-quartile range) as rated by human SMEs along with the word count for the three chatbots (CD, CG, and DS) responses to the different questions.

Domain	Question	Accuracy Ratings Median (IQR)			Number of words		
		CG	CD	DS	CG	CD	DS
C1: Diagnosis and testing	1	8.0 (8–9.25)	8.0 (7–10)	7.5 (5.75–9.25)	189	158	299
	2	10.0 (5–10)	9.0 (7.5–9.25)	10.0 (9.25–10)	304	194	606
C2: Causes and aggravating factors	3	9.5 (5.75–10)	7.5 (5–9.25)	10.0 (9.75–10)	334	146	542
	4	9.0 (7.75–10)	8.0 (7–10)	9.5 (8.75–10)	318	191	572
C3: Symptoms and associated issues	5	8.5 (7–9)	8.5 (7.5–9)	9.0 (8.75–10)	126	149	330
	6	8.5 (7–10)	9.5 (7–10)	9.5 (8–10)	296	201	447
	7	9.0 (6.5–10)	8.5 (7.5–10)	9.0 (7.25–10)	245	169	538
C4: Treatment options	8	9.0 (7.25–10)	8.0 (6.75–9.25)	10.0 (7.25–10)	337	172	673
	9	7.5 (3.5–9)	7.0 (5.75–9.25)	10.0 (8.5–10)	209	210	728
	10	8.0 (5–10)	8.5 (6.5–10)	10.0 (8.5–10)	295	192	687
	11	7.0 (4.75–8)	6.5 (3.75–9)	8.0 (5.5–10)	268	180	664
C5: Management and prognosis	12	8.5 (7–9)	6.0 (5–9)	10.0 (9–10)	219	217	639
	13	9.0 (7.25–9.25)	8.0 (5–8.5)	10.0 (9.75–10)	222	232	613
	14	9.0 (7.25–10)	8.0 (6.5–8.25)	10.0 (9–10)	326	202	693

The AOI index comprises five dimensions and the inter-rater reliability analysis yielded a combined ICC value of 0.47 (moderate agreement). Only one (Clarity and Specificity), achieved moderate agreement with an ICC of 0.58, representing 20% of the rated dimensions. The remaining four (Factual Accuracy, Corroboration, Consistency, and Relevance) all demonstrated poor agreement with ICC values below 0.4, representing 80% of the AOI dimensions. All three AI-based chatbots demonstrated high accuracy (median AOI scores 8–10) across most clinical domains. Among the three AI-based chatbots, DS consistently achieved high accuracy across all domains, with scores frequently reaching 10. CD also demonstrated high accuracy, though some responses fell into the moderate range, particularly in C5-Management and prognosis (Question 12: median = 6, IQR = 5–9) and C4-Treatment options (Question 11: median = 6.5, IQR = 3.75–9). Similarly, CG also exhibited high accuracy, with occasional moderate accuracy observed in specific instances such as C4-Treatment options (Question 9: median = 7.5, IQR = 3.5–9, and Question 11: median = 7.0, IQR = 4.75–8).

Domain-wise responses for C1-Diagnosis and testing, C2-Causes and aggravating factors, and C3-Symptoms and associated issues showed consistently high accuracy across all the three AI-based chatbots. In contrast, for domains C4-Treatment options and C5-Management and prognosis, some responses were in the moderate accuracy range. Notably, in the C4-Treatment options domain (Questions 9 and 11), CG and CD exhibited median scores within the moderate range, while DS maintained high accuracy (Table 2). Heatmap visualization for the median accuracy and empathy scores are presented in Supplementary Table 1. Comparison of accuracy and empathy across clinical domains are presented in Supplementary Table 2.

Pearson correlation analysis revealed weak, non-significant relationships between SME accuracy ratings and response word count across all three chatbots: CG (*r* = 0.1, *p* > 0.05), CD (*r* = −0.2, p > 0.05), and DS (*r* = 0.2, p > 0.05).Similarly, correlation analysis revealed weak, non-significant relationships between SME empathy ratings and response word count across all three chatbots: CG (*r* = −0.1, p > 0.05), CD (*r* = −0.3, p > 0.05), and DS (r = −0.2, p > 0.05).

For SMEs ratings of empathy, the combined ICC value was 0.62 (moderate). All three AI-based chatbots predominantly exhibited moderate empathy (score = 1) across most domains and questions, with limited instances of high empathy (score = 2) and several instances of no empathy (score = 0). Among the three AI-based chatbots, CD demonstrated two instances of high empathy (score = 2), followed by DS with one instance of high empathy (score = 2). CG displayed the most instances of no empathy (score = 0) (Table 3). High empathy (score = 2) was relatively rare, observed only in the CD for C1-Diagnosis and testing (Question 1: median = 2, IQR = 1–2) and C3-Symptoms and associated issues (Q6: median = 2, IQR = 1.75–2). DS was assessed by SMEs as highly empathetic in C5-Management and prognosis (Question 14: median = 2, IQR = 1–2), yet the BERT-based empathy detection model assigned it a non-empathetic score (0). Most responses across all three AI-based chatbots fell in the moderate empathy (score = 1) range. The CG and DS AI-based chatbots frequently received moderate empathetic scores (1) from SMEs despite being assigned non-empathetic scores (0) by the AI model as seen in Table 4. The BERT-based empathy detection model showed discrepancies with the SME panel on several responses, reflecting differences between automated and SME assessments. Mismatch in the AI model output with the SME ratings of the chatbots responses were observed for highly empathetic (*n* = 1) and moderately empathetic (*n* = 23) classifications. However the AI model output and SME ratings matched for highly empathetic (n = 2), moderately empathetic (n = 2), and not empathetic (*n* = 18) classifications in some instances.

**Table 3:** Domain-wise distribution of empathy scores (median and inter-quartile range) as rated by human SMEs along with the word count for the three chatbots (CD, CG, and DS) responses to the different questions.

Domain	Question	Empathy ratings median (IQR)			Number of words		
		CG	CD	DS	CG	CD	DS
C1: Diagnosis and testing	1	0.5 (0–1)	0.5 (0–1)	0.5 (0–1)	189	158	299
	2	1 (1–1.25)	1 (1–1.25)	1 (1–1.25)	304	194	606
C2: Causes and aggravating factors	3	0 (0–1.25)	0 (0–1.25)	0 (0–1.25)	334	146	542
	4	1 (0.75–1)	1 (0.75–1)	1 (0.75–1)	318	191	572
C3: Symptoms and associated issues	5	1 (0–1)	1 (0–1)	1 (0–1)	126	149	330
	6	0.5 (0–1.25)	0.5 (0–1.25)	0.5 (0–1.25)	296	201	447
	7	1 (0.75–1)	1 (0.75–1)	1 (0.75–1)	245	169	538
C4: Treatment options	8	1 (1–1.25)	1 (1–1.25)	1 (1–1.25)	337	172	673
	9	0.5 (0–1)	0.5 (0–1)	0.5 (0–1)	209	210	728
	10	0.5 (0–1)	0.5 (0–1)	0.5 (0–1)	295	192	687
	11	0.5 (0–1)	0.5 (0–1)	0.5 (0–1)	268	180	664
C5: Management and prognosis	12	0.5 (0–1)	0.5 (0–1)	0.5 (0–1)	219	217	639
	13	0 (0–0.25)	0 (0–0.25)	0 (0–0.25)	222	232	613
	14	0 (0–1)	0 (0–1)	0 (0–1)	326	202	693

**Table 4:** Empathy level of responses generated by three chatbots (CD, CG, and DS) as scored by AI-based empathy detection model (BERT) compared with SME ratings. The grey-highlighted cells indicate matches between AI model scores and SME evaluations of empathy in the responses of the different chatbots.

Empathy level	LLM	AI model score	Human SME Median (IQR)	Domain	Question No.
Highly empathetic	CD	2	2 (1–2)	C1	1
	CD	2	2 (1.75–2)	C3	6
	DS	0	2 (1–2)	C5	14
Empathetic	CG	0	1 (1–1.25)	C1	2
	CD	0	1 (0.75–2)	C1	2
	DS	0	1 (0.75–1)	C1	1
	DS	0	1 (0.75–2)	C1	2
	CG	0	1 (0.75–1)	C2	4
	CD	1	1 (1–2)	C2	4
	DS	0	1 (0–2)	C2	3
	DS	0	1 (0.75–1.25)	C2	4
	CG	0	1 (0–1)	C3	5
	CG	0	1 (0.75–1)	C3	7
	CD	0	1 (0.75–1)	C3	5
	CD	1	1 (0.75–1)	C3	7
	DS	0	1 (1–1)	C3	5
	DS	0	1 (0.75–1)	C3	6
	DS	0	1 (0.75–1.25)	C3	7
	CG	0	1 (1–1.25)	C4	8
	CD	0	1 (0.75–1.25)	C4	8
	CD	0	1 (0–1.25)	C4	9
	CD	0	1 (0.75–1.25)	C4	10
	CD	1	1 (0.75–1)	C4	11
	DS	0	1 (0–1)	C4	9
	DS	0	1 (0–1.25)	C4	10
	DS	0	1 (0.75–1.25)	C4	11
	CD	0	1 (0.75–1.25)	C5	12
	CD	1	1 (0–1)	C5	13
	DS	0	1 (0.75–2)	C5	12
	DS	0	1 (0.75–1.25)	C5	13

## 4 Discussion and conclusion

### 4.1 Discussion

This study evaluated and compared the accuracy and empathetic quality of responses generated by three AI-based chatbots to frequently asked questions relating to TMDs. Among the three, DS demonstrated the highest accuracy across all domains. SME evaluations indicated that both CD and DS exhibited empathy in their responses. In contrast, the AI-based empathy scoring model identified CD as the most empathetic. The AI-based empathy scoring model failed to score many sentences that were scored by SME as moderately empathetic. Agreement between the AI model and SME was higher (∼70%) for responses deemed as highly empathetic. The findings from this study underscore both the potential and current limitations of AI chatbots in patient education for TMD. While factual accuracy remains high, the delivery of human-like empathy is inconsistent.

The question selection process in this study was grounded in qualitative research principles and followed two rounds of iterative refinement. It may be argued that the limited number of questions (*n* = 14) could restrict generalizability to the broader TMD population. However, the questions were selected through SME consensus to capture the most commonly expressed patient concerns within a specific clinical environment. From an initial pool of frequent patient queries, a representative set of questions (n = 14) was finalized to ensure broad coverage of key concerns. Furthermore, categorizing them into the five clinical domains helped to avoid redundancy. This relatively small number of questions reduced the cognitive load for the SME raters and kept the evaluation task manageable. Moreover, framing the questions under defined domains provided a structured, patient-centered approach to assessing chatbot responses. Rephrasing the selected questions to reflect how patients might articulate their concerns in real-life ensured that the interactions were representative of non-expert usage**.**

The need for greater consultation with experts and professionals when evaluating chatbots has been recently stressed [25]. The inclusion of SMEs from multiple specialties both in the process of question development (*n* = 4) and in rating (*n* = 8) is a distinct contribution of this study. The involvement of multiple specialists in question development ensured representation of diverse clinical perspectives, prioritization of contextually relevant problems, and alignment with real-world patient encounters within the regional clinical setting. While previous evaluations of AI chatbots in dentistry have predominantly relied on single-speciality assessments [9,11,12], this study integrates multi-specialty SME judgment (orthodontics, prosthodontics, oral radiology, and oral and maxillofacial surgery) to evaluate AI chatbot performance in the context of TMD. This multi-domain SME approach is particularly critical in TMD, where subspecialty perspectives significantly influence clinical decision making.

The present study further expanded the scope of recent investigations in the TMD domain by evaluating a greater number of AI chatbots. Three chatbots (CG, CD, and DS) were selected based on their popularity and accessibility over the past year and a half, with the basic (free) versions used for consistency. Although studies examining user preferences between free and paid conversational chatbot versions are limited, there is a tendency among majority of users to favor free models, primarily due to their accessibility and cost-effectiveness. Default chatbot settings rather than optimized configurations during prompting were used to ensure that results reflect real-world experience rather than theoretical maximum performance. This ecological validity strengthens the practical relevance of study findings.

Of the free versions selected for this study, CG remains the most visited AI platform globally, with approximately 4.7 billion monthly visits [26]. CG3.5 is trained on an Azure AI supercomputing infrastructure to predict and generate text based on preceding context. Its development is based on supervised fine-tuning with human-generated dialogues, followed by reinforcement learning from human feedback to align responses with human preferences [27]. CD ranks among the top ten most widely used AI tools worldwide and has gained notable attention for its advanced reasoning capabilities and emphasis on ethical and safety frameworks. CD variants build upon the classic transformer backbone but introduce enhancements for handling very long contexts (up to 200,000 tokens), and processing within these transformer layers [28]. DS, the most recent entrant, has rapidly gained prominence, marked by a steep rise in user interest and adoption [29]. DeepSeek R1, is grounded in the decoder-only transformer architecture. Compressing key-value representations to reduce memory footprint, and a mixture-of-experts design that activates only a subset of expert sub-networks per token to enhance efficiency are key innovations of the DS model [30].

Once the chatbot responses for the TMD-related queries were obtained, accuracy was evaluated using predefined criteria (factual accuracy, corroboration, consistency, clarity/specificity, and relevance) within a validated framework [11]. Previous studies assessing dental-specific chatbot queries did not report rater reliability or have relied on consensus resolution methods [9,11]. In contrast, independent evaluations in the present study minimized consensus bias and enabled assessment of inter-rater variability. While a prior study employing two SMEs from the same specialty reported high inter-rater reliability [12], the findings of this study differed. Only the clarity / specificity dimension achieving moderate reliability (ICC = 0.58) and likely benefited from observable textual features such as organization, comprehensibility, and level of detail that SME raters could assess. In contrast, poor agreement on factual accuracy (ICC < 0.4) suggests that SME raters varied in their knowledge bases and interpretation of evidence-based standards for TMDs, while low reliability for corroboration reflects subjective judgment about the adequacy of supporting rationale. Similarly, consistency and relevance showed minimal agreement probably because raters applied varying clinical priorities when judging how well chatbot responses addressed questions. In addition to the differences arising from a relatively large number of SME raters (*n* = 8) from four different specialities, the observed low ICC scores in this study likely reflect inherent challenges in evaluating information on a clinical condition for which high-quality evidence remains limited. The absence of calibration training, standardized reference materials, and anchor examples prior to rating may have also possibly contributed to the lower inter-rater reliability.

All three chatbots (CG, CD, and DS) produced responses with high accuracy, with DS demonstrating consistently strong performance across all clinical domains. Accuracy in the C1-Diagnosis and testing, C2-Causes and aggravating factors, and C3-Symptoms and associated issues domains was consistently high accuracy across chatbots, likely reflecting the clarity and standardization of established clinical [31] and imaging criteria [32] for TMD diagnosis. In contrast, greater variability was observed in the C4-Treatment options, and C5-Management and prognosis domains, consistent with the ongoing ambiguities in TMD management. While conservative approaches are widely accepted as first-line care, controversies surround the use of modalities such as botulinum toxin and occlusal splints. For instance, high-quality studies published in the same year report both effectiveness [33] and ineffectiveness [34] of botulinum toxin for TMD-related pain. The off-label status and regulatory constraints surrounding botulinum toxin further complicate consensus. Evidence for occlusal splints is similarly equivocal. Studies report insufficient evidence regarding the effectiveness of occlusal interventions for managing symptoms [35], lack of preventive benefit from orthodontics [36], and no advantages over other interventions [37]. Whereas, meta-analyses have reported moderate to low-quality evidence supporting certain splints [38], with splints demonstrating comparable efficacy to manual therapy in reducing pain and quality of life [39,40].

It is likely that experts from diverse specialties have applied distinct clinical lenses for the five clinical domains leading to the differing expectations for accurate responses. Beyond specialty-specific biases, research shows divergences exist between postgraduate students and seasoned practitioners in their diagnostic and therapeutic approaches to TMD, underscoring the need for standardized education [41]. All of these uncertainties are likely to have contributed to the low inter-rater agreements observed in specific clinical domains in this study and highlight the inherent difficulty in assigning objective accuracy ratings to the TMD condition.

The median accuracy ratings in this study were generally moderate to high, consistent with prior reports evaluating chatbot responses to dental specialty queries [[9], [10], [11], [12]]. For instance, chatbot responses to endodontic questions demonstrated generally accurate information delivery, though performance declined when addressing advanced diagnostic or procedural details [9]. Similarly, when evaluating queries related to orthodontics, multiple chatbots (ChatGPT, Google Bard, and Microsoft Bing) showed comparable performance in providing evidence-based information, although the authors emphasized the critical need for expert oversight in clinical applications [12]. In the context of TMDs, Kula et al. reported that ChatGPT showed promise as an adjunct educational resource for informed decision-making [21].

This study provides among the first comprehensive assessments in dentistry by jointly evaluating the accuracy and empathetic tone of chatbot responses. Additionally, the evaluation of the DS chatbot represents a novel contribution of this study. Although the mean word count of DS was significantly higher than other chatbots, only weak correlations between word count and accuracy ratings were seen. This suggests that SMEs prioritized response content quality over response length, valuing concise responses equivalently to longer ones, provided they adequately addressed the TMD-related question. The consistently high overall performance of DS may therefore be attributed to differences in model architecture and domain-optimization, enabling more direct and clinically aligned responses to TMD-related queries.

### 4.2 Innovation

Empathy is the capacity to recognize and respond to another's emotional state and encompasses cognitive, emotional, and compassionate dimensions. “Artificial empathy” refers to an AI model's ability to mimic these human-like responses. Existing dental AI research largely emphasizes diagnostic accuracy or informational completeness [[9], [10], [11], [12]], neglecting the communicative and relational elements essential to patient-centered care. By treating empathy as a distinct metric alongside accuracy and completeness, this study addresses a key gap in understanding how AI chatbots may operate not only as information sources but also as communication partners. The development and testing of a BERT-based empathy assessment framework in this study offers a novel approach to evaluating the affective dimensions of AI-generated responses to clinical queries. This methodological contribution is especially timely as AI chatbots are increasingly being integrated into patient-facing healthcare platforms.

Any AI model requires a large training dataset, which is unavailable specifically for TMD. While not using domain specific dataset has disadvantages, it does have the advantage of showing that the empathy model developed is independent of domain and can be of more general use. In this study, even though the model developed was trained on empathy gradation based on mental health related sentences, it could successfully detect highly empathetic sentences in the TMD domain. Based on the EPITOME framework, empathy is categorized into emotional reactions, interpretations, and explorations, with gradation in empathy (no empathy, moderate empathy, and high empathy), rather than merely “empathetic” or “non-empathetic”. The EPITOME-trained BERT model is structurally capable of detecting cognitive empathy (through interpretations and explorations) and affective empathy (through emotional reactions). It may partially capture compassionate intent when expressed as emotional concern, though action-oriented support behaviors may not always be detected if they lack explicit emotional or interpretive language. The model is not designed to detect surface politeness or reassurance strategies, which may contribute to perceived supportiveness but fall outside the empathy framework used for annotation.

While validated instruments such as the consultation and relational empathy (CARE) [42] measure and the Jefferson Scale of Empathy [43] exist for assessing clinician empathy in traditional patient-clinician interactions, these tools were developed and validated specifically for healthcare providers in face-to-face clinical encounters. Given the absence of a validated empathy scale designed for AI-generated clinical responses, and recognizing the fundamental differences between human and AI communication modalities, a pragmatic approach was deemed necessary in this study. Therefore, in addition to utilizing the EPITOME dataset annotated on a 3-point scale (0–2) for training the empathy detection AI model, the use of SMEs ratings on the same 3-point scale (0–2) provided a consistent framework for validating model performance.

Unlike earlier empathy detection models (*e.g.,* LSTM, RoBERTa) that were trained and tested only on homogeneous datasets (*e.g.,* Facebook-derived Empathetic Dialogues), the AI model in this study was trained on a geographically unverified, crowd-sourced dataset, which also improves generalizability. The performance of the BERT-based empathy detection system was validated against ratings provided by SMEs. The model achieved a validation accuracy of 0.74, which, while moderate, was the highest obtained in this study.

Based on the SMEs ratings, DS and CD appear more empathetic than CG, specifically in the C5-Management and prognosis domain. The developed model demonstrated high concordance with SME assessments for responses rated as “highly empathetic” (correctly identifying 2 out of 3 sentences, Table 4). However, alignment dropped for low-empathy responses, with only 4 of 28 chatbot-generated “low empathy” responses matching expert consensus. The higher average word count of DS did not correlate with responses with greater empathy and CD demonstrated the most instances of high and moderate empathy, with some degree of alignment between AI model and SME scores. DS and CG often received non-empathetic ratings from the AI model, despite SME evaluators perceiving the responses as empathetic. This pattern suggests that the novel AI model developed underestimated empathetic responses compared to SME evaluations. While this discrepancy appears significant, the wide interquartile ranges in SME ratings suggest intrinsic subjectivity in empathy evaluation even among trained professionals. This empirically demonstrates that even expert consensus on empathy is unstable and challenge assumptions that empathy represents a reliably measurable construct with objective ground truth.

The AI model developed likely detects only explicit markers of empathy, such as keywords or sentence structures, while missing out on subtler contextual cues. Discrepancies were most frequent for moderately empathetic responses, highlighting the inherent ambiguity between ‘empathetic’ and ‘neutral’ language and underscore the complexity of empathy detection and limitations of the AI model developed in this study. This technical limitation is a reflection of a broader conceptual challenge regarding empathy in clinical communication. Empathy in clinical communication exists on a continuum rather than in discrete categories, and what constitutes appropriate empathy may vary depending on clinical context, patient preferences, and cultural communication norms.

Beyond the technical considerations, empathy modelling carries significant ethical implications. Artificial empathy in healthcare chatbots raises ethical challenges related to patient trust [44]. While simulated empathy can improve comfort, it risks deception, as patients may mistake algorithmic responses for genuine compassion. Additionally, higher empathy scores are not always desirable. In diagnostic contexts requiring clear communication excessive emotional cushioning may impair comprehension. If a healthcare chatbot softens responses to maximize perceived empathy, it may inadvertently downplay urgency. Empathy must be calibrated to clinical context, lower in situations demanding clarity and urgency, and higher in contexts requiring emotional support and validation. Chatbots that incorporate empathy and user emotion recognition are no doubt important, but equally critical is to establish universally accepted ethical standards for their effective integration into healthcare [[45], [46], [47]]. Once this is possible, AI-based chatbots could be successfully used as a pre-appointment triage tool to provide patients with preliminary information about TMD or supplement patient education materials. This is particularly important in TMD care, where the biopsychosocial nature of the condition means that empathetic communication is not merely a courtesy but a therapeutic component.

This study's results also carry important implications for how AI chatbots used in clinical settings are regulated and certified. As these tools become more prevalent in direct patient interactions, their evaluation should go beyond simple accuracy measures [48]. The research findings from this study suggest that regulatory standards for medical AI chatbots should employ dual tracks, with one assessing clinical accuracy, and evidence-base using objective standards, and another evaluating communication quality and empathy using specialized experts and validated patient-experience frameworks. Despite their ability to generate empathetic responses, chatbots remain limited in their ability to interpret and meaningfully probe a user's lived experience, in contrast to human clinicians [49]. The inconsistency observed in empathy evaluations also highlights the necessity for uniform certification standards and continuous monitoring even after AI chatbots are deployed.

### 4.3 Limitations

This study has several limitations. The single-shot prompting structure used in the study restricts chatbot's ability to express empathy. In single-turn interactions, empathetic language is typically confined to brief introductory phrases (*e.g.,* “I'm sorry to hear that,” Table 5) before transitioning to factual explanations. However, empathy in human communication typically unfolds across multiple conversational exchanges. The absence of human-generated responses for comparison is another study limitation. While it was beyond the scope of this study to collect human-generated responses, establishing a benchmark human empathetic response for each query could help contextualize chatbot performance relative to real clinician-patient communication in future studies.

**Table 5:** Exemplars of sentences scored by the AI model as highly empathetic (score 2), and moderately empathetic (score1) and non- empathetic (score 0).

AI score	Sentence
2	I'm sorry to hear about your TMD diagnosis - I know this condition can be quite uncomfortable. Let me explain what TMD means and involves.
2	I'm sorry you're experiencing headaches and migraines - yes, TMD can definitely cause these issues. Let me explain the connection.
1	Would you like me to share some specific ways to help manage this TMD-related ear pain? There are several techniques that might bring you relief.
1	Yes, stress can definitely make TMD symptoms worse, and it sounds particularly relevant given your work situation. I'm sorry you're dealing with this challenging combination. Let me explain the stress-TMD connection:
1	Would you like to discuss specific management strategies that might work well for your situation?
1	Orthodontic treatment to correct your bite (occlusion) might help with TMD symptoms, but it's important to understand that it's not always a guaranteed cure. Here's what you should know:
0	Temporomandibular disorder (TMD) refers to a group of conditions that affect the jaw joint (temporomandibular joint or TMJ) and the muscles that control jaw movement,
0	This can cause pain, difficulty chewing, clicking or popping sounds, and even ear or facial discomfort,
0	It's a common condition, and while it can be frustrating, the good news is that there are many effective treatments to help manage your symptoms,

Another limitation is the variability in SME ratings, reflected in low inter-rater reliability for both accuracy and empathy assessments. This highlights the subjectivity inherent in evaluating both informational accuracy and empathetic quality. The low ICC values do not merely reflect rater disagreement, but also epistemic uncertainty in the construct being measured, especially for empathy and clinical appropriateness in TMD. Although ratings were averaged for analysis, it must be kept in mind that averaging SME ratings under such conditions yields a descriptive consensus, not a gold standard. Readers should exercise caution when interpreting median scores as definitive judgments of overall chatbot quality.

There are differences between perceived empathy and actual therapeutic empathy. Perceived empathy reflects patients' subjective experience of feeling heard and validated, whereas therapeutic empathy refers to empathetic communication that actually contributes to clinical outcomes, therapeutic alliance, and patient wellbeing. The empathy ratings in this study were provided by SMEs rather than patient experience metrics. While patient-reported empathy ratings can provide insight into the user experience dimension, the focus of this study was on outcomes that require expert judgment. Future research examining patient experiential satisfaction would complement this study, but address a distinct set of research questions.

The limited agreement between human and AI empathy assessments (especially for mid-range responses), underscores challenges for AI models in evaluating nuanced expressions of empathy. Instances where the BERT empathy model's predictions diverged from SME ratings should not be interpreted as the model ‘disagreeing’ with SME judgment, but rather as construct misalignment. The BERT empathy model detects learned textual patterns that may correlate with perceived empathy but do not necessarily align with SME evaluations of clinical appropriateness or therapeutic empathy. Its moderate validation accuracy (∼0.74) likely approaches the ceiling for text-based prediction given the subjectivity of empathy ratings and the inability of textual features alone to capture the full dimensionality of empathetic communication. It is important to recognize that AI models lack genuine emotional comprehension and can only simulate empathetic language.

Lastly, only baseline accessible technology was evaluated in this study and model capabilities differ across versions. AI models are frequently updated, and outputs may vary over time. The results reported here reflect model performance at the time of testing, and future or updated versions could respond differently. This temporal variability is an inherent limitation associated when evaluating AI models.

Future research should address these limitations through several complementary approaches. First, multistage prompting strategies or conversational dialogue structures should be adopted to better capture dynamic expressions of empathy. Second, mixed-methods designs combining quantitative metrics with patient interviews would provide more comprehensive assessments of emotional impact from the patient perspective. Third, studies should include human-generated responses as benchmarks for comparison. Fourth, fine-tuning AI models to optimize both domain-specific knowledge and contextually appropriate empathetic responses should be prioritized, with evaluation of patient perceptions and real-world clinical utility. Finally, given that large language models may encode training data biases and that TMD disproportionately affects women, systematic evaluation of demographic-based variations in response quality, and empathy is warranted.

Despite these limitations, study findings contribute to understanding AI chatbot performance with implications for developing accurate and empathetic systems across specialties, including psychology, primary care, and chronic disease management, where communication is essential for patient engagement and outcomes. With progress in natural language processing, emotion-detection systems, and adaptive algorithms, the utility and responsiveness of AI chatbots also continues to improve. Emerging evidence suggests multimodal platforms can now synthesize data from sources beyond just textual exchanges by integrating voice, visual, and biometric data, enabling a more comprehensive patient evaluation [50]. In future, validated empathy-aware AI chatbots could be integrated into pre-visit triage workflows to provide emotionally responsive initial guidance, deployed in automated patient education and counselling platforms, or incorporated into AI-human hybrid communication models where AI augments clinician responses with empathetic language suggestions.

### 4.4 Conclusions

AI chatbots show promise in providing accurate information regarding TMDs, but their ability to convey empathy remains limited. The observed differences in accuracy and empathy among the three AI chatbots examined are based on a limited dataset and should therefore be interpreted with caution. Current AI chatbots represent an intermediate stage of development, demonstrating adequate technical proficiency while remaining constrained in addressing the humanistic dimensions of patient care. Although empathy detection models may inform future development, significant challenges in empathetic communication persist.

## CRediT authorship contribution statement

**Maryam Shehab:** Methodology, Funding acquisition, Formal analysis, Data curation. **Tanmoy Bhattacharjee:** Writing – original draft, Methodology, Formal analysis, Conceptualization. **Hisham Mohammed:** Writing – review & editing, Writing – original draft, Methodology, Investigation. **Prasad Nalabothu:** Investigation. **Moosa Alhuwaitat:** Investigation. **Keyvan Moharamzadeh:** Investigation. **Jahanzeb Chaudhry:** Investigation. **Nader Nabil Fouad:** Methodology, Investigation. **Abdel Rahman Tawfik:** Investigation. **Sabarinath Prasad:** Writing – review & editing, Writing – original draft, Visualization, Validation, Supervision, Resources, Project administration, Methodology, Investigation, Funding acquisition, Formal analysis, Data curation, Conceptualization.

## Informed consent

Not applicable.

## Ethical approval

The study was reviewed and approved by the Mohammed Bin Rashid University (MBRU IRB-2024-613) and regional (DSREC-Sr-12/2024_02) ethics committees under the exempt category.

## Declaration of generative AI and AI-assisted technologies in the writing process

During the preparation of this work the author(s) used Claude 4 Sonnet (Anthropic) as a writing assistance tool in the second revision of the manuscript in order to refine sentence structure, and enhance the overall clarity of the manuscript. After using Claude 4 Sonnet (Anthropic), the author(s) reviewed and edited the content as needed and take(s) full responsibility for the content of the published article.

## Funding

Author M.S. has received research support from the MBRU resident research fund supported the work.

## Declaration of competing interest

None of the authors have any financial and non-financial conflict of interests.

## Appendix A Supplementary data

Supplementary Data S1. Codes for text classification implementations covering (a) Data Preparation and Preprocessing, (b) Bag-of-Words Classification, (c) Long Short-Term Memory (LSTM) Neural Network, and (d) BERT-based Sequence Classification. Image 1

Supplementary Table 1 shows domain-wise accuracy and empathy scores (median [IQR], rated by human SMEs) and word count for three chatbots (CD, CG, DS). Heatmap colour gradient ranges from red (low) to green (high). Domains: C1 = Diagnosis & testing; C2 = Causes & aggravating factors; C3 = Symptoms & associated issues; C4 = Treatment options; C5 = Management & prognosis. Image 2

Supplementary Table 2 : Comparison of accuracy and empathy across clinical domains (C1: Diagnosis and testing, C2: Causes and aggravating factors, C3: Symptoms and associated issues, C4: Treatment options, and C5: Management and prognosis) using Friedman's test with Dunn's post-hoc pairwise comparisons and 95% confidence intervals. Image 3
