# Supplementary material for: Accuracy and empathy of AI-based conversational chatbots in response to temporomandibular dysfunction related queries
Source: PEC Innov. 2026 Feb 23;8:100463. doi: 10.1016/j.pecinn.2026.100463 (PMC12992975; doi:10.1016/j.pecinn.2026.100463)
Supplement: Supplementary file 1 — Supplementary Data S1. Codes for text classification implementations covering (a) Data Preparation and Preprocessing, (b) Bag-of-Words Classification, (c) Long Short-Term Memory (LSTM) Neural Network, and (d) BERT-based Sequence Classification. [file mmc1.docx]

Supplementary data on Codes used for Text Classification implementations including

(a)Data Preparation and Preprocessing (Code 1),

(b) Bag-of-Words Classification Approach (Code 2),

(c) implement a Long Short-Term Memory (LSTM) neural network for sequential text processing(Code 3) and ,

(d) Python implementation utilized the BERT (Bidirectional Encoder Representations from Transformers) architecture for advanced sequence classification (Code 4)

**Code 1**

*% Load the dataset*

filename = "EmpatheticConversations.csv";

data = readtable(filename, TextType="string");

*% Display first few rows*

head(data)

*% Convert Empathy column to categorical*

data.Empathy = categorical(data.Empathy);

classNames = categories(data.Empathy);

*% Create stratified train-validation split (80-20)*

cvp = cvpartition(data.Empathy, Holdout=0.2);

dataTrain = data(training(cvp), :);

dataValidation = data(test(cvp), :);

*% Separate text and labels for training set*

textDataTrain = dataTrain.utterance;

YTrain = dataTrain.Empathy;

*% Separate text and labels for validation set*

textDataValidation = dataValidation.utterance;

YValidation = dataValidation.Empathy;

**Code 2**

*% Create bag-of-words model from training documents*

bag = bagOfWords(documentsTrain);

*% Remove words that appear less than 2 times*

bag = removeInfrequentWords(bag, 2);

*% Remove empty documents and get their indices*

[bag, idx] = removeEmptyDocuments(bag);

*% Remove corresponding labels for empty documents*

YTrain(idx) = [];

*% Display bag-of-words information*

bag;

*% Extract word count matrix for training*

Xtrain = bag.Counts;

ytrain = YTrain;

*% Prepare test data*

dataTest = data(test(cvp), :);

textDataTest = dataTest.utterance;

documentsTest = preprocessText(textDataTest);

*% Encode test documents using the trained bag-of-words*

Xtest = encode(bag, documentsTest);

ytest = dataTest.Empathy;

*% Train multiclass classification model using ECOC*

Mdllda = fitcecoc(Xtrain, ytrain);

**Code 3**

*% Create word encoding from training documents*

enc = wordEncoding(documentsTrain);

*% Set sequence length for padding/truncation*

sequenceLength = 15;

*% Convert documents to sequences for training*

XTrain = doc2sequence(enc, documentsTrain, Length=sequenceLength); XTrain(1:5)

*% Convert documents to sequences for validation*

XValidation = doc2sequence(enc, documentsValidation, Length=sequenceLength);

*% Define LSTM network architecture*

inputSize = 1;

embeddingDimension = 50;

numHiddenUnits = 80;

numWords = enc.NumWords;

numClasses = numel(categories(YTrain));

layers = [ ...

sequenceInputLayer(inputSize) wordEmbeddingLayer(embeddingDimension, numWords) lstmLayer(numHiddenUnits, OutputMode="last") fullyConnectedLayer(numClasses) softmaxLayer];

*% Configure training options*

options = trainingOptions("adam", ...

InputDataFormats="CTB", ...

MiniBatchSize=16, ...

GradientThreshold=2, ...

Shuffle="every-epoch", ...

ValidationData={XValidation,YValidation}, ... Plots="training-progress", ...

Metrics="accuracy", ...

Verbose=false);

*% Train the LSTM network*

net = trainnet(XTrain, YTrain, layers, "crossentropy", options);

*% Test on new data*

documentsNew = preprocessText(reportsNew);

XNew = doc2sequence(enc, documentsNew, Length=sequenceLength);

*% Make predictions*

scores = minibatchpredict(net, XNew, InputDataFormats="CTB"); labelsNew = scores2label(scores, classNames)

**Code 4**

# -*- coding: utf-8 -*-

"""

Created on Mon Feb 24 00:57:48 2025

@author: TANMOY

"""

# Import required libraries

import numpy as np

import pandas as pd

import time

import datetime

import gc

import random

from nltk.corpus import stopwords

import re

import torch

import torch.nn as nn

from torch.utils.data import TensorDataset, DataLoader, RandomSampler, SequentialSampler, random_split

from sklearn.model_selection import train_test_split

from sklearn.metrics import classification_report

import transformers

from transformers import BertForSequenceClassification, AdamW, BertConfig, BertTokenizer, get_linear_schedule_with_warmup

# Set device

device = torch.device("cuda:0" if torch.cuda.is_available() else "cpu")

print(device)

# Load data

df = pd.read_csv('Training.csv')

# Load stopwords

sw = stopwords.words('english')

# Text cleaning function

def clean_text(text):

text = text.lower()

text = re.sub(r"[^a-zA-Z?.!,¿]+", " ", text) # Keep only letters and punctuation

text = re.sub(r"http\S+", "", text) # Remove URLs

html = re.compile(r'<.*?>')

text = html.sub(r'', text) # Remove HTML tags

punctuations = '@#!?+&*[]-%.:/();$=><|{}' + "'`" + '_'

for p in punctuations:

text = text.replace(p, '') # Remove punctuations

text = [word.lower() for word in text.split() if word.lower() not in sw]

text = " ".join(text) # Remove stopwords

emoji_pattern = re.compile("["

u"\U0001F600-\U0001F64F" # Emoticons

u"\U0001F300-\U0001F5FF" # Symbols & pictographs

u"\U0001F680-\U0001F6FF" # Transport & map symbols

u"\U0001F1E0-\U0001F1FF" # Flags (iOS)

u"\U00002702-\U000027B0"

u"\U000024C2-\U0001F251"

"]+", flags=re.UNICODE)

text = emoji_pattern.sub(r'', text) # Remove emojis

return text

# Apply text cleaning

df['text'] = df['text'].apply(lambda x: clean_text(x))

# Extract tweets and labels

tweets = df.text.values

labels = df.target.values

# Load BERT tokenizer

tokenizer = BertTokenizer.from_pretrained('bert-base-uncased', do_lower_case=True)

# Find maximum sentence length

max_len = 0

for sent in tweets:

input_ids = tokenizer.encode(sent, add_special_tokens=True)

max_len = max(max_len, len(input_ids))

print('Max sentence length: ', max_len)

# Tokenize all tweets

input_ids = []

attention_masks = []

for tweet in tweets:

encoded_dict = tokenizer.encode_plus(

tweet,

add_special_tokens=True,

max_length=max_len,

padding='max_length',

return_attention_mask=True,

return_tensors='pt',

truncation=True

)

input_ids.append(encoded_dict['input_ids'])

attention_masks.append(encoded_dict['attention_mask'])

# Convert lists to tensors

input_ids = torch.cat(input_ids, dim=0)

attention_masks = torch.cat(attention_masks, dim=0)

labels = torch.tensor(labels)

# Print example

print('Original: ', tweets[0])

print('Token IDs:', input_ids[0])

# Create TensorDataset

dataset = TensorDataset(input_ids, attention_masks, labels)

# Create train-validation split (80-20)

train_size = int(0.8 * len(dataset))

val_size = len(dataset) - train_size

train_dataset, val_dataset = random_split(dataset, [train_size, val_size])

print('{:>5,} training samples'.format(train_size))

print('{:>5,} validation samples'.format(val_size))

# Create DataLoaders

batch_size = 32

train_dataloader = DataLoader(

train_dataset,

sampler=RandomSampler(train_dataset),

batch_size=batch_size

)

validation_dataloader = DataLoader(

val_dataset,

sampler=SequentialSampler(val_dataset),

batch_size=batch_size

)

# Load BERT model for sequence classification

model = BertForSequenceClassification.from_pretrained(

"bert-base-uncased",

num_labels=3,

output_attentions=False,

output_hidden_states=False

)

model = model.to(device)

# Set up optimizer

optimizer = AdamW(

model.parameters(),

lr=2e-5,

eps=1e-8

)

# Set up training parameters

epochs = 4

total_steps = len(train_dataloader) * epochs

# Create learning rate scheduler

scheduler = get_linear_schedule_with_warmup(

optimizer,

num_warmup_steps=0,

num_training_steps=total_steps

)

# Accuracy calculation function

def flat_accuracy(preds, labels):

pred_flat = np.argmax(preds, axis=1).flatten()

labels_flat = labels.flatten()

return np.sum(pred_flat == labels_flat) / len(labels_flat)

# Time formatting function

def format_time(elapsed):

elapsed_rounded = int(round((elapsed)))

return str(datetime.timedelta(seconds=elapsed_rounded))

# Set random seeds for reproducibility

seed_val = 42

random.seed(seed_val)

np.random.seed(seed_val)

torch.manual_seed(seed_val)

torch.cuda.manual_seed_all(seed_val)

# Training loop

training_stats = []

total_t0 = time.time()

for epoch_i in range(0, epochs):

# Training phase

print("")

print('======== Epoch {:} / {:} ========'.format(epoch_i + 1, epochs))

print('Training...')

t0 = time.time()

total_train_loss = 0

model.train()

for step, batch in enumerate(train_dataloader):

b_input_ids = batch[0].to(device)

b_input_mask = batch[1].to(device)

b_labels = batch[2].to(device)

optimizer.zero_grad()

output = model(

b_input_ids,

token_type_ids=None,

attention_mask=b_input_mask,

labels=b_labels

)

loss = output.loss

total_train_loss += loss.item()

loss.backward()

torch.nn.utils.clip_grad_norm_(model.parameters(), 1.0)

optimizer.step()

scheduler.step()

avg_train_loss = total_train_loss / len(train_dataloader)

training_time = format_time(time.time() - t0)

print("")

print(" Average training loss: {0:.2f}".format(avg_train_loss))

print(" Training epoch took: {:}".format(training_time))

# Validation phase

print("")

print("Running Validation...")

t0 = time.time()

model.eval()

total_eval_accuracy = 0

best_eval_accuracy = 0

total_eval_loss = 0

nb_eval_steps = 0

for batch in validation_dataloader:

b_input_ids = batch[0].to(device)

b_input_mask = batch[1].to(device)

b_labels = batch[2].to(device)

with torch.no_grad():

output = model(

b_input_ids,

token_type_ids=None,

attention_mask=b_input_mask,

labels=b_labels

)

loss = output.loss

total_eval_loss += loss.item()

logits = output.logits

logits = logits.detach().cpu().numpy()

label_ids = b_labels.to('cpu').numpy()

total_eval_accuracy += flat_accuracy(logits, label_ids)

avg_val_accuracy = total_eval_accuracy / len(validation_dataloader)

print(" Accuracy: {0:.2f}".format(avg_val_accuracy))

avg_val_loss = total_eval_loss / len(validation_dataloader)

validation_time = format_time(time.time() - t0)

if avg_val_accuracy > best_eval_accuracy:

torch.save(model, 'bert_model.pt')

best_eval_accuracy = avg_val_accuracy

training_stats.append({

'epoch': epoch_i + 1,

'Training Loss': avg_train_loss,

'Valid. Loss': avg_val_loss,

'Valid. Accur.': avg_val_accuracy,

'Training Time': training_time,

'Validation Time': validation_time

})

print("")

print("Training complete!")

print("Total training took {:} (h:mm:ss)".format(format_time(time.time() - total_t0)))

# Load best model

model = torch.load('bert_model.pt', weights_only=False)

# Test on new data

df_test = pd.read_csv('Sabtest.csv')

df_test['text'] = df_test['text'].apply(lambda x: clean_text(x))

test_tweets = df_test['text'].values

# Tokenize test data

test_input_ids = []

test_attention_masks = []

for tweet in test_tweets:

encoded_dict = tokenizer.encode_plus(

tweet,

add_special_tokens=True,

max_length=max_len,

padding='max_length',

return_attention_mask=True,

return_tensors='pt',

truncation=True

)

test_input_ids.append(encoded_dict['input_ids'])

test_attention_masks.append(encoded_dict['attention_mask'])

test_input_ids = torch.cat(test_input_ids, dim=0)

test_attention_masks = torch.cat(test_attention_masks, dim=0)

# Create test dataset and dataloader

test_dataset = TensorDataset(test_input_ids, test_attention_masks)

test_dataloader = DataLoader(

test_dataset,

sampler=SequentialSampler(test_dataset),

batch_size=batch_size

)

# Make predictions

predictions = []

for batch in test_dataloader:

b_input_ids = batch[0].to(device)

b_input_mask = batch[1].to(device)

with torch.no_grad():

output = model(

b_input_ids,

token_type_ids=None,

attention_mask=b_input_mask

)

logits = output.logits

logits = logits.detach().cpu().numpy()

pred_flat = np.argmax(logits, axis=1).flatten()

predictions.extend(list(pred_flat))

# Save predictions

df_output = pd.DataFrame()

df_output['target'] = predictions

df_output.to_csv('submissionSabTest.csv', index=False)

# Calculate confusion matrix

trueClasstest = df_test.target.values

from sklearn import metrics

confusion_matrix = metrics.confusion_matrix(trueClasstest, predictions)

print(confusion_matrix)

# Process Word document

import docx2txt

my_text = docx2txt.process("document.docx")

print(my_text)

list2 = my_text.split('.')

# Save to CSV

import csv

with open('log.csv', 'w', newline='') as out_file:

writer = csv.writer(out_file)

writer.writerow(['text'])

writer.writerows([[item] for item in list2])

# Process document data

df_test = pd.read_csv('log2.csv')

df_test = df_test.dropna()

df_test['text'] = df_test['text'].apply(lambda x: clean_text(x))

test_tweets = df_test['text'].values

# Tokenize document data

test_input_ids = []

test_attention_masks = []

for tweet in test_tweets:

encoded_dict = tokenizer.encode_plus(

tweet,

add_special_tokens=True,

max_length=max_len,

padding='max_length',

return_attention_mask=True,

return_tensors='pt',

truncation=True

)

test_input_ids.append(encoded_dict['input_ids'])

test_attention_masks.append(encoded_dict['attention_mask'])

test_input_ids = torch.cat(test_input_ids, dim=0)

test_attention_masks = torch.cat(test_attention_masks, dim=0)

# Create dataset and dataloader

test_dataset = TensorDataset(test_input_ids, test_attention_masks)

test_dataloader = DataLoader(

test_dataset,

sampler=SequentialSampler(test_dataset),

batch_size=batch_size

)

# Make predictions on document

predictions = []

for batch in test_dataloader:

b_input_ids = batch[0].to(device)

b_input_mask = batch[1].to(device)

with torch.no_grad():

output = model(

b_input_ids,

token_type_ids=None,

attention_mask=b_input_mask

)

logits = output.logits

logits = logits.detach().cpu().numpy()

pred_flat = np.argmax(logits, axis=1).flatten()

predictions.extend(list(pred_flat))

# Save final predictions

df_output = pd.DataFrame()

df_output['predictions'] = predictions

df_output.to_csv('document_predictions.csv', index=False)
