# Supplementary material for: Accuracy and empathy of AI-based conversational chatbots in response to temporomandibular dysfunction related queries
Source: PEC Innov. 2026 Feb 23;8:100463. doi: 10.1016/j.pecinn.2026.100463 (PMC12992975; doi:10.1016/j.pecinn.2026.100463)
Supplement: Supplementary file 2 — Supplementary Table 1 shows domain-wise accuracy and empathy scores (median [IQR], rated by human SMEs) and word count for three chatbots (CD, CG, DS). Heatmap colour gradient ranges from red (low) to green (high). Domains: C1 = Diagnosis & testing; C2 = Causes & aggravating factors; C3 = Symptoms & associated issues; C4 = Treatment options; C5 = Management & prognosis. [file mmc2.docx]

| **Domain** | **Question** | **Accuracy Ratings**  **Median (IQR)** | | | **Empathy Ratings**  **Median (IQR)** | | | **Number of words** | | |
| --- | --- | --- | --- | --- | --- | --- | --- | --- | --- | --- |
|  |  | **CG** | **CD** | **DS** | **CG** | **CD** | **DS** | **CG** | **CD** | **DS** |
| CI | 1 | 8.0 (8–9.25) | 8.0 (7–10) | 7.5 (5.75–9.25) | 0.5 (0–1) | 2 (1–2) | 1 (0.75–1) | 189 | 158 | 299 |
|  | 2 | 10.0 (5–10) | 9.0 (7.5–9.25) | 10.0 (9.25–10) | 1 (1–1.25) | 1 (0.75–2) | 1 (0.75–2) | 304 | 194 | 606 |
| C2 | 3 | 9.5 (5.75–10) | 7.5 (5–9.25) | 10.0 (9.75–10) | 0 (0–1.25) | 0.5 (0–1) | 1 (0–2) | 334 | 146 | 542 |
|  | 4 | 9.0 (7.75–10) | 8.0 (7–10) | 9.5 (8.75–10) | 1 (0.75–1) | 1 (1–2) | 1 (0.75–1.25) | 318 | 191 | 572 |
| C3 | 5 | 8.5 (7–9) | 8.5 (7.5–9) | 9.0 (8.75–10) | 1 (0–1) | 1 (0.75–1) | 1 (1–1) | 126 | 149 | 330 |
|  | 6 | 8.5 (7–10) | 9.5 (7–10) | 9.5 (8–10) | 0.5 (0–1.25) | 2 (1.75–2) | 1 (0.75–1) | 296 | 201 | 447 |
|  | 7 | 9.0 (6.5–10) | 8.5 (7.5–10) | 9.0 (7.25–10) | 1 (0.75–1) | 1 (0.75–1) | 1 (0.75–1.25) | 245 | 169 | 538 |
| C4 | 8 | 9.0 (7.25–10) | 8.0 (6.75–9.25) | 10.0 (7.25–10) | 1 (1–1.25) | 1 (0.75–1.25) | 1.5 (1–2) | 337 | 172 | 673 |
|  | 9 | 7.5 (3.5–9) | 7.0 (5.75–9.25) | 10.0 (8.5–10) | 0.5 (0–1) | 1 (0–1.25) | 1 (0–1) | 209 | 210 | 728 |
|  | 10 | 8.0 (5–10) | 8.5 (6.5–10) | 10.0 (8.5–10) | 0.5 (0–1) | 1 (0.75–1.25) | 1 (0–1.25) | 295 | 192 | 687 |
|  | 11 | 7.0 (4.75–8) | 6.5 (3.75–9) | 8.0 (5.5–10) | 0.5 (0–1) | 1 (0.75–1) | 1 (0.75–1.25) | 268 | 180 | 664 |
| C5 | 12 | 8.5 (7–9) | 6.0 (5–9) | 10.0 (9–10) | 0.5 (0–1) | 1 (0.75–1.25) | 1 (0.75–2) | 219 | 217 | 639 |
|  | 13 | 9.0 (7.25–9.25) | 8.0 (5–8.5) | 10.0 (9.75–10) | 0 (0–0.25) | 1 (0–1) | 1 (0.75–1.25) | 222 | 232 | 613 |
|  | 14 | 9.0 (7.25–10) | 8.0 (6.5–8.25) | 10.0 (9–10) | 0 (0–1) | 0.5 (0–1) | 2 (1–2) | 326 | 202 | 693 |

Supplementary Table 1: Domain-wise distribution of accuracy and empathy scores (median and inter-quartile range) as rated by human SMEs along with the word count for the three chatbots (CD, CG, and DS) responses to the different questions. Note the heatmap visualization that uses a colour gradient from red (low values) to green (high values) for the median accuracy and empathy scores. C1: Diagnosis and testing, C2: Causes and aggravating factors, C3: Symptoms and associated issues, C4: Treatment options, and C5: Management and prognosis
