# Supplementary material for: Accuracy and empathy of AI-based conversational chatbots in response to temporomandibular dysfunction related queries
Source: PEC Innov. 2026 Feb 23;8:100463. doi: 10.1016/j.pecinn.2026.100463 (PMC12992975; doi:10.1016/j.pecinn.2026.100463)
Supplement: Supplementary file 3 — Supplementary Table 2 : Comparison of accuracy and empathy across clinical domains (C1: Diagnosis and testing, C2: Causes and aggravating factors, C3: Symptoms and associated issues, C4: Treatment options, and C5: Management and prognosis) using Friedman's test with Dunn's post-hoc pairwise comparisons and 95% confidence intervals. [file mmc3.pdf]

Parameter	Domain	Friedman's p-value	Dunn's Test Comparisons	Confidence Interval (CI)			
Accuracy	Combined	<0.0001	CG vs. DS: –52.5, **, p = 0.0014<br>CD vs. DS: –63, ****, p <0.0001<br>CG vs. CD: 10.5, ns, p >0.9999	Lower 95% CI<br>Upper 95% CI	7.088<br>8.109	7.024<br>7.958	8.088<br>8.983
	C1	0.1719		Lower 95% CI<br>Upper 95% CI	7.288<br>9.337	6.315<br>9.310	7.141<br>9.359
	C2	0.2639		Lower 95% CI<br>Upper 95% CI	6.544<br>9.456	6.302<br>8.823	7.846<br>10.03
	C3	0.1643		Lower 95% CI<br>Upper 95% CI	6.876<br>8.874	7.207<br>9.043	7.777<br>9.39
	C4	0.001	CG vs. DS: –23, *, p = 0.0121<br>CD vs. DS: -20.5, *, p = 0.0312<br>CG vs. CD: -2.5, ns, p >0.9999	Lower 95% CI<br>Upper 95% CI	5.773<br>7.977	6.106<br>8.144	7.213<br>9.225
	C5	0.0007	CG vs. DS: -16.5, *, p = 0.0517<br>CD vs. DS: - 22.5, **, p = 0.0035<br>CG vs. CD: 6, ns, p >0.9999	Lower 95% CI<br>Upper 95% CI	6.266<br>8.817	6.152<br>8.014	7.681<br>9.986
Empathy	Combined	<0.0001	CG vs. DS: –52.5, **, p = 0.0014<br>CD vs. DS: -13.5, ns, p>0.9999<br>CG vs. CD: -39, *, p=0.0275	Lower 95% CI<br>Upper 95% CI	0.552<br>0.804	0.844<br>1.12	0.900<br>1.171

Dubai Health - Internal Use

	**C1**	0.1456		Lower 95% CI<br>Upper 95% CI	0.463<br>1.162	0.889<br>1.735	0.575<br>1.300
	**C2**	0.3679		Lower 95% CI<br>Upper 95% CI	0.337<br>1.163	0.526<br>1.349	0.564<br>1.435
	**C3**	0.0784		Lower 95% CI<br>Upper 95% CI	0.464<br>1.035	0.812<br>1.438	0.751<br>1.249
	**C4**	0.0337		Lower 95% CI<br>Upper 95% CI	0.472<br>0.965	0.657<br>1.155	0.725<br>1.275
	**C5**	<0.0001	CG vs. DS: –24, **, p = 0.0016<br>CD vs. DS: -13.5, ns, p=0.154<br>CG vs. CD: -10.5, ns, p=0.3889	Lower 95% CI<br>Upper 95% CI	0.170<br>0.663	0.464<br>1.035	0.879<br>1.537

Supplementary Table 2 : Comparison of accuracy and empathy across clinical domains (C1: Diagnosis and testing, C2: Causes and aggravating factors, C3: Symptoms and associated issues, C4: Treatment options, and C5: Management and prognosis) using Friedman's test with Dunn's post-hoc pairwise comparisons and 95% confidence intervals.

Dubai Health - Internal Use
